# Forced diuresis with matched hydration in reducing acute kidney injury during transcatheter aortic valve implantation (Reduce-AKI): study protocol for a randomized sham-controlled trial

**DOI:** 10.1186/1745-6215-15-262

**Published:** 2014-07-02

**Authors:** Yaron Arbel, Eyal Ben-Assa, Amir Halkin, Gad Keren, Arie Lorin Schwartz, Ofer Havakuk, Eran Leshem-Rubinow, Maayan Konigstein, Arie Steinvil, Yigal Abramowitz, Ariel Finkelstein, Shmuel Banai

**Affiliations:** 1 Department of Cardiology, Tel-Aviv Medical Center affiliated to the Sackler Faculty of Medicine, Tel-Aviv University, 6 Weizman Street, Tel Aviv, Israel

## Abstract

**Background:**

Acute kidney injury (AKI) is observed in up to 41% of patients undergoing transcatheter aortic valve implantation (TAVI) and is associated with increased risk for mortality. The aim of the present study is to evaluate whether furosemide-induced diuresis with matched isotonic intravenous hydration using the RenalGuard system reduces AKI in patients undergoing TAVI.

**Methods/Design:**

Reduce-AKI is a randomized sham-controlled study designed to examine the effect of an automated matched hydration system in the prevention of AKI in patients undergoing TAVI. Patients will be randomized in a 1:1 fashion to the RenalGuard system (active group) versus non-matched saline infusion (sham-controlled group). Both arms receive standard overnight saline infusion and N-acetyl cysteine before the procedure.

**Discussion:**

The Reduce-AKI trial will investigate whether the use of automated forced diuresis with matched saline infusion is an effective therapeutic tool to reduce the occurrence of AKI in patients undergoing TAVI.

**Trial registration:**

Clinicaltrials.gov: NCT01866800, 30 April 30 2013.

## Background

Acute kidney injury (AKI) is a frequent complication of coronary angiography, and is associated with unfavorable outcomes including major cardiovascular events, renal replacement therapy, prolonged hospitalization, and early death [1,2]. Recently, two randomized controlled trials have demonstrated that furosemide-induced diuresis with matched isotonic intravenous hydration using the RenalGuard system (PLC Medical Systems, Milford, Massachusetts, USA) [3,4] reduces AKI in high-risk patients undergoing coronary procedures.

Elderly patients undergoing TAVI have a high prevalence of chronic kidney disease (CKD) and therefore are at increased risk to develop AKI (Table 1). In recent reports, AKI has been observed in up to 41% of patients undergoing TAVI [5-11] and was associated with a four times higher post-procedural mortality [11-14]. ‘RenalGuard’ is a Conformité Européenne (CE)-approved system that is being used worldwide. The objective of this randomized sham-controlled study is to assess the beneficial effect of treating TAVI patients with this system in reducing the occurrence of AKI.

**Table 1:** **Definition of acute kidney injury post transcatheter aortic valve implantation (TAVI) according to the VARC-2 classification **[15]**]**

**Stage 1**	Increase in serum creatinine to 150 to 199% of baseline
	**OR**
	Increase of ≥0.3 mg/dl (26.4 mmol/L)
	**OR**
	Urine output <0.5 ml/kg/h for >6 but <12 h
**Stage 2**	Increase in serum creatinine to 200 to 299% of baseline
	**OR**
	Urine output <0.5 ml/kg/h for >2 but <24 h
**Stage 3**	Increase in serum creatinine to ≥300% of baseline
	**OR**
	Increase of serum creatinine of ≥4 mg/dL (354 mmol/L) with an acute increase of at least 0.5 mg/dl (44 mmol/l)
	**OR**
	Urine output <0.3 ml/kg/h for >24 h
	**OR**
	Anuria for >12 h

## Methods/Design

We describe a single-center, double-blinded, randomized sham-controlled trial that is being conducted at the Tel Aviv Medical Center, Tel Aviv, Israel.

### Transcatheter aortic valve implantation procedures

All patients will undergo transfemoral aortic valve implantation per standard clinical practice. Briefly, the femoral artery will be accessed with the standard endovascular technique, thereafter the procedure involves advancing a large catheter (18 Fr) through the aortic arch, retrogradely crossing the aortic valve, following by balloon valvuloplasty and stent-valve implantation. During the procedure, the patients will receive analgesics and anxiolytics as per protocol.

Post-procedural pharmacotherapy, sheath removal, and deployment of hemostatic devices will be left to the discretion of the attending cardiologists. Following treatment in the catheterization laboratory, medical treatment throughout hospitalization and follow-up treatment will be left to the discretion of the treating physician managing patient care on the hospital wards. Specific agents added or withdrawn will be made by treating physicians and will not be influenced by the study team.

### RenalGuard system

**Figure 1:**
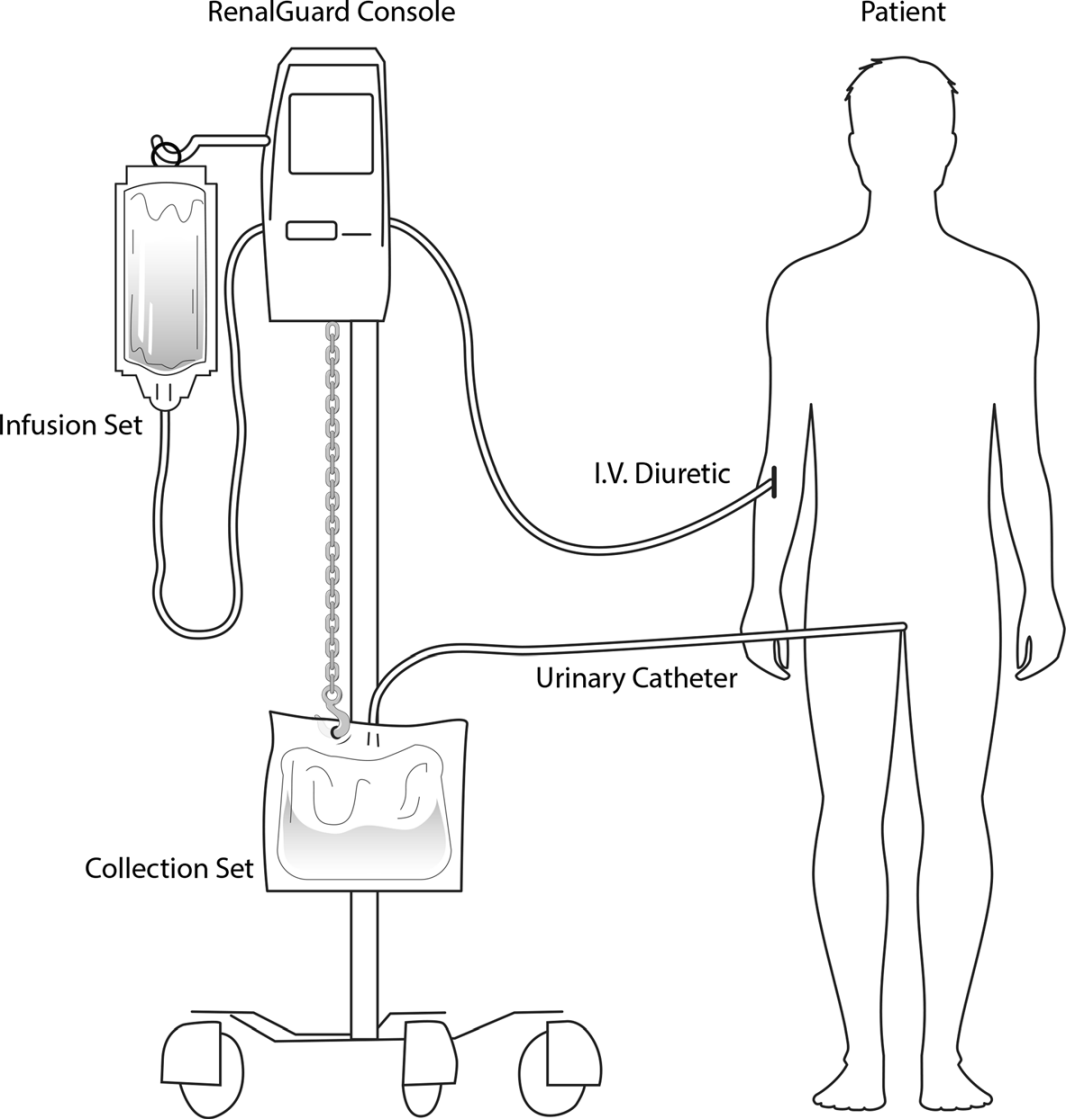
The ‘RenalGuard’ system.

The RenalGuard system (PLC Medical Systems, Milford, Massachusetts, USA) is a CE approved system that is being used worldwide. It includes a computer system that weighs the urine of the patient, calculates the urine rate (ml/min) and infuses matched IV normal saline into the patient, maintaining the initially programmed fluid balance. In our medical center, the RenalGuard system is routinely used for high-risk patients undergoing coronary procedures (Figure 1).

### Study population

A total of 220 patients will be randomized. Eligible patients must be planned for elective TAVI and all must have an estimated glomerular filtration rate (eGFR) below 60 ml/min/1.73 m^2^. We limited the number of inclusion and exclusion criteria in order to simulate real-life patients.

### Inclusion criteria

The inclusion criteria are as follows:

1. subject who is able and willing to give an informed consent is ≥65 years old,

2. is undergoing planned transfemoral TAVI due to severe aortic stenosis, and

3. has calculated eGFR below 60 ml/min/1.73 m2 (using Modification of Diet in Renal Disease (MDRD) formula).

### Exclusion criteria

The exclusion criteria are as follows:

1. history of acute coronary syndrome in the past 30 days,

2. history of congestive heart failure with left ventricular ejection fraction <30% or exacerbation in the past 30 days,

3. current dialysis treatment,

4. known furosemide hypersensitivity, or

5. contraindications to placement of a Foley catheter in the bladder.

### Randomization and blinding

**Figure 2:**
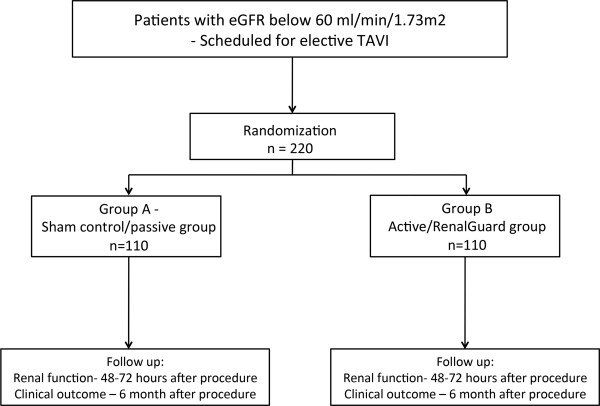
Study flow chart.

At the time of enrollment, signed informed consent will be obtained as per the Tel Aviv Medical Center institutional ethical board standards. After signing the informed consent, patients will be randomized in a 1:1 fashion using closed envelopes. They will be randomized to the active use of RenalGuard system versus using passive use of the RenalGuard system using normal saline 0.9% infusion (sham-controlled group) (Figure 2). The RenalGuard system will be covered before, during and after the procedure with a bag in order to allow effective blinding. All participants will remain blinded throughout the 6-month study period. The interventional cardiologist conducting the TAVI procedure is also blinded to the study treatment arm. The supervising physician, core labs, the biostatisticians performing the analysis, the members of the Clinical Event Committee (CEC), as well as the members of the Data Safety Monitoring Board (DSMB) are blinded to treatment assignment until study completion and after the database has been unlocked.

### Study protocol

#### Pre-procedural evaluation

After enrollment, patients will undergo the following baseline procedures: physical examination and medical interview; cardiac echocardiogram assessing systolic and diastolic function, as well as valvular function; endothelial function using the EndoPat™ system, a non-invasive method for assessing endothelial function; carotid artery ultrasound and doppler including Intima Media Thickness calculations; and blood and urine analysis as described below.

All participating patients will receive a standard pre-procedural hydration treatment plan consisting of an infusion of isotonic saline at a rate of 0.5 to 1 mL/kg per hour, starting 12 h prior to the procedure and continuing for 12 h after contrast administration. In addition, 1,200 mg of N-acetyl-cysteine will be administered orally twice daily the day before and the day of the procedure.

**Figure 3:**
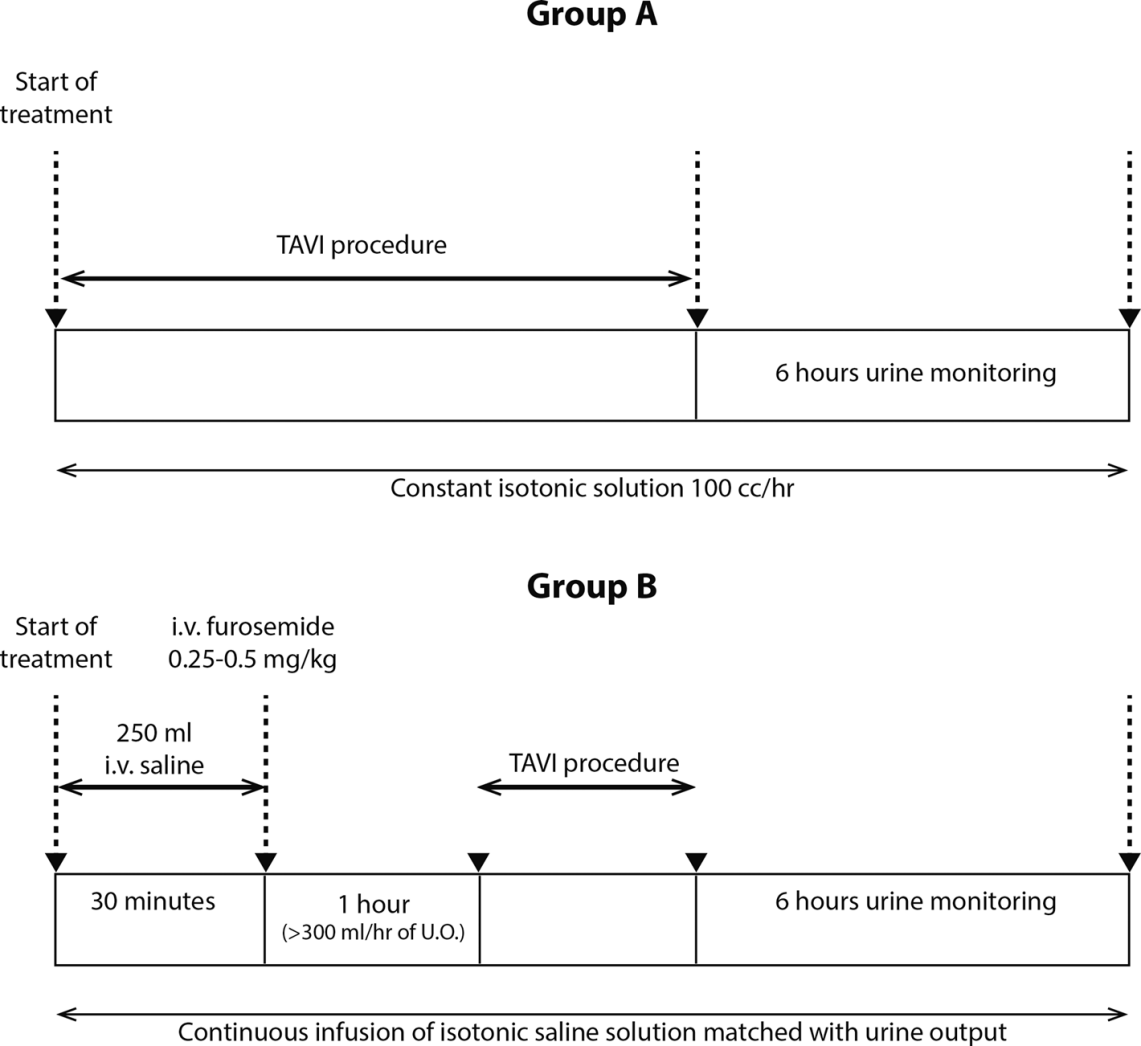
Treatment algorithms of the sham-controlled/passive RenalGuard group (group A) and the active RenalGuard group (group B).

Each patient will be randomized to one of the following treatment strategies:

**Group 1** - Sham-controlled Group (Figure 3A). The patients will be connected to the RenalGuard System that will not be activated (passive mode).

Before the procedure: A standard 18 to 22 gauge catheter will be inserted into a peripheral vein of the arm and a standard Foley catheter will be placed in the bladder for urine collection (as performed routinely for all TAVI procedures). The intravenous (IV) line and the urinary catheter will be connected to the RenalGuard System. The RenalGuard System will be set to 0% match; that is, the urine rate and volume will be measured, but it will not deliver matched IV normal saline. The system will be computed to deliver continues IV normal saline at a rate of 100 cc/h (100 cc/h is the routine average fluid infusion rate in TAVI procedure) during the procedure and up to 6 h after the procedure. Neither a routine IV bolus of saline nor a routine IV furosemide will be given before the procedure.

During the procedure: The urine and infusion volumes will be measured by the RenalGuard system throughout the catheterization procedure and for 6 h after the last contrast dose. Additional IV fluids can be added as needed according to the clinical decision of the anesthesiologist or the attending physician.

We will analyze the quantity and quality of urine during the duration of the procedure.

**Group 2** - Active Group (Figure 3B). Treatment as group 1 and in addition, the RenalGuard system will be activated (active mode).

Before the procedure: A standard 18 to 22 gauge catheter will be inserted into a peripheral vein of the arm and a standard Foley catheter will be placed in the bladder for urine collection (as performed routinely for all TAVI procedures). The IV line and the urinary catheter will be connected to the RenalGuard System. The RenalGuard System will be set to 100% match; that is, the urine rate and volume will be measured and the system will deliver volume of IV normal saline that is matched to the volume of urine produced by the patient. An initial IV bolus (250 ml) of normal saline over 30 min will be administered approximately 90 min before the procedure; in patients with heart failure, the volume of the bolus can be reduced to 150 ml. Furosemide will then be administered as a single intravenous bolus of 0.25 to 0.5 mg/kg at the physician’s discretion. When a urine output rate >300 ml/h will be achieved, the patients will be sent to the catheterization laboratory.

During the procedure: Additional doses of furosemide (up to a maximal cumulative dose of 2.0 mg/kg) will be given in cases where the urine output is below 300 ml/h during treatment. Matched hydration will continue throughout the catheterization procedure and for 6 h after the last contrast dose. Additional IV fluids can be added as needed according to the clinical decision of the anesthesiologist or the attending physician. We will analyze the quantity and quality of urine during the duration of the procedure.

### Blood and urinary samples

Blood samples will be taken at baseline (on admission); before the procedure; and at 6, 12, 24, 48, and 72 h post-procedure. An 18-gauge cannula will be placed in an antecubital vein for blood sampling. Blood sample analyses will be performed using reagents, calibrators and control materials from Bayer Diagnostics (Berkshire, England) on the ADVIA 1650. At baseline each Patient will provide 40 cc of blood for the following blood tests: full chemistry including lipid levels, thyroid function, BNP, NT-BNP, HbA1c, uric acid, and glucose levels; complete blood count; inflammatory biomarker (hs-CRP, fibrinogen, IL-6, IL-1, MMP, Lp-PLA2, procalcitonin, IL-10, IL-35, TNFa, AchE, and cholinergic status); renal function markers including creatinine, NGAL (neutrophil gelatinase-associated lipocalin), FGF23, and Cystatin-C; and endothelial function markers including I-CAM, V-CAM, superoxide dismutase ADMA, and oxidized LDL. Serum and plasma samples will be frozen for future tests.

Urine samples will be taken at baseline (before the procedure), and after 24 h.

Samples will be taken for basic urinalysis, creatinine, albumin, microalbumin, and electrolytes.

### Study objectives

Reduce-AKI is a prospective, randomized, double-blind, sham-controlled clinical trial of the safety and effectiveness of the RenalGuard system in patients with severe aortic stenosis and eGFR <60 ml/min/1.73 m2 undergoing elective TAVI.

### Primary endpoint

The primary endpoint is reduction of acute kidney injury (stage 1 or above) at 48 to 72 h. Serum creatinine level will be measured prior to TAVI and at 48 to 72 h post-procedure. AKI will be defined as stage 1, 2 or 3 according to VARC-2 AKI classification [15] (Table 1).

### Secondary endpoints

The secondary endpoints are to assess whether the RenalGuard system can reduce major adverse clinical events (MACE) defined as a composite of all-cause mortality, myocardial infarction, AKI, 30 day readmission rate, and dialysis. Another secondary endpoint is to assess whether the RenalGuard system can lower 30-day readmission rate and 30-day congestive heart failure exacerbation rate.

Other secondary objectives are to determine whether endothelial function assessment can predict AKI using the EndoPat™ system, to determine whether chronic statins treatment offers any clinical benefit in preventing contrast induced nephropathy, to determine if carotid Doppler analysis can predict AKI occurrence and to determine the predictive value of different biomarkers in assessing the clinical outcome in TAVI patients.

### Ethics, informed consent

The study protocol was approved by the Tel Aviv Sourasky Medical Center Institutional Review Board/ Ethics (Helsinki) Committee, approval number: 0111-13-TLV. Oral and written informed consent from the patient will be obtained prior to inclusion.

### Adverse events

After the procedure, participants are assessed every day until discharge and then at 30 days, 3 months, and 6 months (Table 2). At each of these follow-up visits, the occurrence of adverse events is evaluated by medical interview and review of medical records for every patient.

**Table 2:** Flow chart of the anticipated procedures during the study follow-up

	**Baseline**	TAVI^* ^**procedure**	**Hospitalization**	**Discharge**	**1 month ± 7 days**	**6 months ± 14 days**
Informed consent	**√**					
Full medical history	**√**					
Physical examination	**√**		**√**	**√**	**√**	**√**
Blood tests	**√**	**√**	**√**	**√**	**√**	**√**
Endothelial function	**√**					
Urinary analysis	**√**	**√**	**√**			
Carotid doppler	**√**					
12 lead ECG^†^	**√**			**√**		
Cardiac echo	**√**			**√**	**√**	**√**
Adverse events assessment		**√**	**√**	**√**	**√**	**√**

*TAVI - transcatheter aortic valve implantation; ^†^ECG – electrocardiogram.

The sign **√** indicates the timing at which the procedures listed above are performed.

### Safety

The RenalGuard system is approved for coronary angiography. Since all patients undergoing TAVI undergo urinary catheterization with a Foley catheter, our major safety issue is related to fluid overload. We will assess this issue by evaluating both in-hospital and 30-day readmission rate for congestive heart failure exacerbation, using patient medical records and medical interviews. In addition, we will evaluate all other safety criteria according to the VARC-2 criteria for TAVI procedures [15].

### Interim analysis

An independent DSMB is chartered to monitor and evaluate patient safety to identify any clinically relevant trends, and to recommend whether the study should continue. The DSMB review will occur after 30 randomized patients have completed the 30-day follow-up and after approximately 25%, 50%, and 75% of the cohort has completed their six-month follow-up. An interim analysis for efficacy is planned after 50% of the cohort is enrolled.

### Power analysis

The study is powered to detect a 50% reduction in AKI between the groups. The following assumptions and hypotheses correspond to the primary objective:

A = 40% of participants in the sham-controlled group will develop AKI.

B = 20% of participants in the active group will develop AKI.

Hypotheses:

H0: A = B

H1: A ≠ B

Type I error rate = 5% (2-sided) with power = 80%

Drop out/lost to follow –up, rate = 5%

Under the assumption that AKI is relatively common (40%) after TAVI, and that the RenalGuard system has been shown to reduce the incidence of AKI by up to 70% [3,4], we estimated that if there will be a 50% reduction in AKI in the RenalGuard group, the number of patients needed in each group to attain 80% power with an alpha of 0.05, is 92. We plan on recruiting 110 patients in each group (220 total) in order to assure achieving statistical power.

### Statistical analysis

Standard statistical analyses will be used to compare the aforementioned endpoints in both study arms. We will compare the incidence of AKI and any clinical differences between the groups. Between-group comparisons of clinical endpoints, biomarkers, and imaging data will be performed using the Mann-Whitney U, independent Student’s t tests, or chi square test according to the distribution of variables. All values will be expressed as medians and interquartile ranges for non-normally distributed continuous variables and as mean and standard error for normally distributed variables.

The statistical analysis of the primary endpoint will performed and presented following the intent-to-treat (ITT) principle. A second set of analysis will be performed using a per-protocol population which will include all enrolled patients who completed the procedure and had a 6-month follow-up. Reported *P* values will be two-sided, and *P* <0.05 will be considered as statistically significant. All analyses will be performed using SPSS statistical software.

## Discussion

The Reduce-AKI trial will investigate whether the use of forced diuresis with matched saline infusion using the RenalGuard system will reduce the occurrence of AKI in patients undergoing elective transfemoral TAVI.

AKI following TAVI is an independent predictor of short- and long-term outcome [6,11] In previously published studies, the incidence of AKI in patients undergoing TAVI ranged from 12% to 41% [6,9-11] with dialysis required in 1.5 to 10% [6,16].

The reasons for the increased risk of AKI in patients undergoing TAVI include elder population with multiple comorbidities, peri-procedural bleedings, hemodynamic instability, distal embolization and the use of nephrotoxic drugs.

Hydration is the cornerstone of AKI prevention. It increases the intravascular blood volume, suppresses the renin-angiotensin-aldosterone system, and promotes dilution and rapid evacuation of contrast media [4,9,17].

Furosemide may reduce the nephrotoxic effect of contrast media, first by increasing the urine flow and thus diluting the contrast media, and second by blocking tubular sodium reabsorption in the loop of Henle, thus reducing tubular workload and concomitant oxygen requirement.

The use of furosemide alone is controversial since it reduces the effective circulating volume and prostaglandin mediated vasodilation, and may lead to dehydration as a result of increased urine output [18,19]. However, when combining hydration with furosemide, studies have shown improved outcomes [3,4].

The RenalGuard system combines all the above methods for prevention of AKI and hydration with forced diuresis with furosemide, while preventing dehydration or fluid overload.

Recently, two randomized controlled trials [3,4] have demonstrated that furosemide-induced diuresis with matched isotonic intravenous hydration using the RenalGuard system reduces AKI in high-risk patients undergoing coronary procedures by up to 71%. In the present study, we chose to evaluate the effect of the RenalGuard system on patients with reduced eGFR since they are at the highest risk for AKI [6,7,20]. In most TAVI patients present with an eGFR below 60 ml/min/1.73 m2, the low eGFR is mostly due to their advanced age (above 80 years). The inclusion/exclusion criteria were very lenient in order to simulate ‘real life’ patients. Therefore, we believe that the Reduce-AKI study will demonstrate a reduction of AKI in this high-risk patient population.

The Reduce-AKI trial has several limitations that need to be discussed. The relatively small sample size will not answer the clinical benefit associated with the RenalGuard system based upon hard cardiovascular events. However, if a reduction of AKI is demonstrated, we feel that it will add valuable information, which might have an effect on the treatment approach to these patients. Second, we will need to conduct a cost-benefit analysis if we find a positive result in order to evaluate its application in clinical practice.

### Trial status

The trial is ongoing. Currently 41 patients have been recruited.

## Abbreviations

AKI: acute kidney injury; CE: Conformité Européenne; CEC: clinical event committee; CKD: chronic kidney disease; DSMB: data safety monitoring board; eGFR: estimated glomerular filtration rate; ITT: intent-to-treat; MACE: major adverse clinical events; MDRD: Modification of Diet in Renal Disease TAVI, transcatheter aortic valve implantation; VARC-2: Valve Academic Research Consortium-2.

## Competing interest

A.F. serves as a consultant and proctor of both Medtronic Cardiovascular and Edwards Lifesciences. All other authors declare that they have no competing interests.

## Authors’ contributions

YA and EBA are joint head investigators for the study, they conceived the study, participated in the design, coordination, and data collection of the study, and wrote the first draft of the manuscript. AH participated in the design and data collection of the study, and revised the manuscript. GK: participated in the design of the study and critical revision and final approval of the manuscript. ALS: participated in data collection and statistical analysis of the study. OH: participated in data collection and analysis, manuscript revising and final approval of the manuscript. ELR: participated in manuscript writing, data analysis and final approval of manuscript. MK: participated in data collection and analysis, and revised the manuscript. AS: participated in data collection, statistical analysis, and final approval of the manuscript. YA: participated in data collection and manuscript writing and revision. AF: is in charge of all the clinical aspects of TAVI procedures, he participated in the conception and design of the study and final approval of the manuscript. SB: is the principal investigator of the study, he participated in the conception and design of the study, interpretation of data, critical revision and final approval of the manuscript. All authors read and approved of the final version of the manuscript.
